# Prediction and secondary prevention of preeclampsia from the perspective of public health management – the initiative of the State of Rio de Janeiro

**DOI:** 10.61622/rbgo/2024EDT03

**Published:** 2024-05-27

**Authors:** Antonio Braga, Penélope Saldanha Marinho, Marcos Nakamura-Pereira, José Carlos Peraçoli, Cláudia Mello

**Affiliations:** 1 Universidade Federal Fluminense Niterói RJ Brazil Universidade Federal Fluminense, Niterói, RJ, Brazil.; 2 Universidade de Vassouras Vassouras RJ Brazil Universidade de Vassouras, Vassouras, RJ, Brazil.; 3 Secretária de Estado de Saúde do Rio de Janeiro Rio de Janeiro RJ Brazil Secretária de Estado de Saúde do Rio de Janeiro, Rio de Janeiro, RJ, Brazil.; 4 Universidade Federal do Rio de Janeiro Rio de Janeiro RJ Brazil Universidade Federal do Rio de Janeiro, Rio de Janeiro, RJ, Brazil.; 5 Fundação Oswaldo Cruz Instituto Nacional de Saúde da Mulher, da Criança e do Adolescente Fernandes Figueira Rio de Janeiro RJ Brazil Instituto Nacional de Saúde da Mulher, da Criança e do Adolescente Fernandes Figueira, Fundação Oswaldo Cruz, Rio de Janeiro, RJ, Brazil.; 6 Departamento de Obstetrícia e Ginecologia Faculdade de Medicina de Botucatu Botucatu SP Brazil Departamento de Obstetrícia e Ginecologia, Faculdade de Medicina de Botucatu, Botucatu, SP, Brazil.

At least in the last two decades, Brazil has established policies to reduce maternal mortality (MM). Despite international agreements, in which the Millennium Development Goals stand out, as well as proposed actions by the *Rede Cegonha* Program, it was impossible to achieve minimum MM indicators, even thought around 90% of these deaths are preventable.^(1)^

This scenario worsened with the COVID-19 pandemic, resulting in a high maternal mortality (MM) rate in Brazil. Therefore, it established a great challenge to achieve the respective Sustainable Development Goals (SDGs), defined as an MM rate of 30 maternal deaths for every 100,000 live births by 2030.^(2–6)^ In Rio de Janeiro state, the MM scenario is drastic. Historically, the MM rate in the state of Rio de Janeiro was consistently above the SDGs’ agreement, and in 2021, it occupied the worst raking in the Southeast, with the 4th worst position in Brazil.^(3,7)^

There is an increase in indirect causes of MM, primarily owing to advanced maternal age associated with underlying conditions at the time of these pregnancies (particularly hypertension, diabetes, and obesity). However, MM due to direct obstetric causes is still predominant in Rio de Janeiro state. Preeclampsia/eclampsia is the most prevalent cause of MM in Rio de Janeiro state, even as observed in Brazil and Latin America, where hypertension and obstetric hemorrhage compete for the leading cause.^(7)^

The prevention of MM caused by preeclampsia/eclampsia presents a particular challenge once the management of this condition depends on healthcare coordination and continuity of care. It involves primary health care, especially in its prenatal component; the stratification of high-risk pregnancy and appropriate referral to specialized prenatal care, through an official patient regulation system; pre-hospital emergency units and hospitals, notably maternity wards; and finally, returning to primary health care for continued assessment, given the immediate and late risks of hypertension.

Historically, many interventions have been studied to decrease the risk of preeclampsia/eclampsia, such as rest, salt restriction in the diet, supplementation with vitamin C, D, E, omega-3, folic acid, and enoxaparin prescription. Unfortunately, none of these interventions provided evidence of protective action and should not be applied in clinical practice.^(8,9)^

At the current scientific evidence, three strategies are proven to reduce the risk of preeclampsia/eclampsia and should be widely implemented in obstetric care. The first strategy is the recommendation of physical exercise practice during pregnancy; the second is universal supplementation of calcium during prenatal care in populations with low calcium intake; and the last is the prescription of acetylsalicylic acid in cases of well-established obstetric risk for preeclampsia/eclampsia.^(8,9)^

Physical exercise is characterized by planned, structured, and repetitive body movements to improve one or more components of physical fitness, one of the essential elements of a healthy lifestyle. Therefore, obstetrician-gynecologists and other healthcare professionals who provide obstetric care should encourage their patients to continue or initiate exercise as a necessary component of optimal health. It is known that physical exercise helps reduce the risk of obesity, diabetes, hypertension, and mental health problems in adults. A sedentary lifestyle is the fourth most prominent risk factor for death worldwide, and new evidence has emerged showing the importance of physical exercise during pregnancy.^(10,11)^

Systematic review with meta-analysis showed a significantly reduced risk of preeclampsia in pregnant women who performed aerobic exercise for 30 to 60 minutes, 2 to 7 times a week, compared to sedentary women.^(12)^ Another systematic review with meta-analysis including normal-risk pregnant women, normal weight, singleton pregnancies, who practiced aerobic exercise for 35-90 minutes, 3-4 times a week, did not find an association with a higher risk of preterm birth or a reduction in mean gestational age of childbirth.^(13)^.

These studies show that women who habitually practiced vigorous-intensity aerobic activities or were physically active before pregnancy can continue these activities during pregnancy and postpartum. Physical activity during pregnancy is associated with minimal risks and many benefits for most women. However, some adaptations to exercise routines may be necessary due to changes in the maternal organism and fetal needs.

Since Belizan's original studies in the 1980s, calcium has been the most studied supplement for preventing preeclampsia. Calcium supplementation was the second most used procedure in preeclampsia prevention studies.^(14)^ Since 2011, the World Health Organization has recommended calcium supplementation to prevent preeclampsia in all pregnant women on a low-calcium diet (< 900 mg calcium/day) as well as for pregnant at high risk for developing preeclampsia.^(15)^

Epidemiological investigations reveal that low dietary calcium intake is a risk factor for preeclampsia, a significant contributor to obstetric morbidity and mortality.^(16)^ Brazilian Institute of Geography and Statistics data show that the average calcium intake in the Brazilian population is 476 mg/day, slightly higher in the Southeast region (505 mg/day) but still insufficient.^(17)^

Systematic review and meta-analysis of 13 studies that compared high-dose calcium supplementation (≥ 1 g/day) versus placebo in 15,730 women found a 55% reduction in the occurrence of preeclampsia in the group with calcium supplementation compared to placebo [relative risk (RR) = 0.45, 95% confidence interval (CI) = 0.31 to 0.65; I² = 70%].^(18)^

The effect was more effective for pregnant at higher risk of developing preeclampsia (depending on the following clinical factors: nulliparity, maternal age over 35 years, history of hypertension or diabetes during or outside pregnancy). In this high-risk group, it was observed a reduction of 78% in preeclampsia, although this may be due to the limited number of patients with this condition (five trials, 587 women included: mean RR 0.22, 95% CI 0.12 to 0.42).^(18)^

Likewise, when analyzing the effect of supplementation in pregnant women on a low-calcium diet (< 900 mg calcium/day), eight randomized clinical trials including 10,678 women found a 64% reduction in the occurrence of preeclampsia (RR: 0 .36; 95% CI: 0.20-0.65).^(18)^

There is no consensus regarding the dose of calcium supplementation to prevent preeclampsia. Although the World Health Organization recommends calcium supplementation at a dose of between 1,500 and 2,000 mg daily, divided into three doses, its use is not widely implemented in Brazil for pregnant women with low dietary calcium intake to reduce the risk of preeclampsia.

A recent publication presented the results of two independent randomized clinical trials that evaluated the impact of calcium supplementation in 22,000 nulliparous from India and Tanzania (countries with a population with low calcium intake), comparing the effect of different doses of calcium, 500mg versus 1,500mg, at the study outcome (occurrence of preeclampsia). The study concluded that low-dose calcium supplementation was not inferior to high-dose supplementation regarding preeclampsia, highlighting the possibility of a lower dose of supplemented calcium to achieve a protective outcome against preeclampsia.^(19)^

Thus, calcium supplementation during pregnancy reduces the risk of preeclampsia, especially in those at high risk of developing preeclampsia or on a diet low in calcium. Globally, the evidence also points to the possibility of a 20% reduction in maternal morbidity and mortality risk.

Since supplementation with calcium carbonate has minimal side effects, wide acceptability, and is a low-cost supplement available from the National List of Essential Medicines of the Brazilian National Healthcare System - SUS (it is estimated that each 1,250mg tablet of calcium carbonate costs US$0.02 or US$10.00/patient for treatment throughout all the pregnancy) and is associated with a 50% reduction in the occurrence of preeclampsia in the general obstetric population, the incorporation of its universal use during pregnancy should be widely recommended. Furthermore, considering that the Brazilian population in general, and the Southeast in particular, has a low calcium intake and that, in this specific group, calcium supplementation is even more effective.^(14,20)^

**Table 1:** List of calcium found in food sources

Food	Amount	Calcium (mg)
Almond	200 g	508
Curd	100 g	490
Tofu	¼ cup	430
Sesame	100 g	417
Broccoli, raw leaves	100 g	400
Instant-cook oats	100 g	392
Cress	200 g	336
Skimmed milk	200 mL	250
Yogurt	200 mL	240
Açai	200 g	236
Fresh Minas cheese	1	205
Kale cabbage	2 tablespoon	164
Cooked spinach	4 tablespoon	160
Cooked broccoli	100 g	130
Raw dry lentils	100 g	107
Prune	100 g	62
Boiled chicken egg	100 g	54

Evidence from the last decade has consistently indicated that the administration of low doses of acetylsalicylic acid during prenatal care is associated with a reduction in the risk of preeclampsia in a high-risk population, with an associated benefit of reduction in the risk of preterm birth, intrauterine growth restriction, and maternal and perinatal mortality in this same high-risk group.^(21–23)^

The United States Preventive Services Task Force recently reviewed the prevention of preeclampsia using acetylsalicylic acid and found evidence of a reduction in risk of preeclampsia (pooled RR: 0.85, 95% CI: 0.75-0.95; 16 studies; I^2^ = 0%) with the use of low-dose aspirin in individuals at increased risk (n = 14,093). Among individuals at increased risk of preeclampsia who received low-dose aspirin (n = 13,619), pooled estimates provided evidence of reduced risk of preterm birth (RR: 0.80, 95% CI: 0.67-0. 95; 13 studies; I^2^ = 49%), and restricted intrauterine growth (RR: 0.82, 95% CI: 0.68-0.99; 16 studies; I^2^ = 41.0%) in individuals at increased risk of preeclampsia (n = 14,385). There was also a reduction in perinatal mortality (RR: 0.79, 95% CI: 0.66-0.96; 11 studies; I^2^ = 0%) in individuals at increased risk of preeclampsia (n = 13,860). Maternal complications of preeclampsia (e.g., eclampsia or death) occurred rarely in studies and could not be appropriately assessed.^(24)^

On the other hand, the analysis of 21 randomized clinical trials (n = 26,757; 14 good quality, seven fair quality) to evaluate maternal, perinatal, and postnatal developmental harms showed no evidence of damage resulting from daily acetylsalicylic acid use in low doses during pregnancy. Hemorrhagic complications was uncommon and pooled results were not statistically significant for occurrence of placental abruption (pooled RR: 1.15, 95% CI: 0.76-1.72; I^2^ = 25%; 10 trials; n = 24,970), postpartum hemorrhage (pooled RR: 1.03, 95% CI: 0.94 −1.12; I^2^ = 0%; 9 trials; n = 23,133) or fetal intracranial bleeding (pooled RR: 0.90, 95% CI: 0.51-1.57; I^2^ = 19%; 6 trials; n = 23,719). Similarly, evidence on long-term child development outcomes in offspring from in-utero exposure to low doses of aspirin has been limited. Follow-up data from the most extensive study, the Collaborative Low-dose Aspirin Study in Pregnancy (CLASP),^(25)^ reported no differences in physical or developmental outcomes (e.g., motor development, height, weight, or hospital visits) in newborns at 12 and 18 months. Finally, no differences were found in studies reporting other rare perinatal injuries (e.g., congenital anomalies or malformations).^(24)^

The results of preventing pre-eclampsia with higher doses of acetylsalicylic acid (150 mg) are encouraging. As soon as new studies crystallize these observations, it would be ideal for the pharmaceutical industry to commercialize this dosage in order to offer prevention to pregnant women at risk of pre-eclampsia. Although cost-effective has already been established, the incorporation of biophysical methods (such as the uterine artery pulsatility index) or biochemical methods (such as PAPP-A and PLGF dosage) in the calculation of obstetric risk for the use of acetylsalicylic acid has not yet been incorporated in the state of Rio de Janeiro, which will focus its policy on preventing pre-eclampsia using only risk factors from the anamnesis and physical examination for this assessment. The best is the enemy of the good.

Therefore, the use of acetylsalicylic acid in low doses is recommended for pregnant women at high risk for preeclampsia (level of evidence A) as it reduces the incidence of preeclampsia by 17% (which can reach a reduction of 67% in early-onset preeclampsia) and in 14% fetal or neonatal death. The recommended dose is 100 mg (low dose) at night from the 12th week onwards (preferably before the 16th week, however, could be started up to the 20th week) and maintained until the 36th week of pregnancy. ^(23–28)^

The early identification of patients at risk for preeclampsia through clinical risk markers can help in the implementation of preventive measures to avoid or delay the onset of the disease or even reduce its severity.^(21–26)^

Primary health care providers have the critical role of starting qualified and early prenatal care, which will allow the identification of risk factors for preeclampsia and the implementation of preventive measures. Furthermore, they can coordinate the care of these patients, who will also be monitored in a high-risk service.

The following chart 1 shows the primary clinical risk markers related to preeclampsia and the indication for preeclampsia prophylaxis with acetylsalicylic acid.^(9)^

**Chart 1:** Clinical markers

Risk stratification	Clinical and/or Obstetric Presentation
High-Risk Only one risk factor is needed to initiate acid acetylsalicylic prophylaxis	History of preeclampsia, mainly accompanied by adverse outcomes
	Multiple gestation
	Obesity (Body mass index> 30)
	Chronic arterial hypertension
	Type 1 or 2 diabetes
	Kidney disease
	Autoimmune diseases (Ex: Systemic lupus erythematosus, antiphospholipid syndrome)
	Pregnancy resulting from assisted reproduction
Moderate Risk ≥ 2 factors of risk are needed to initiate acid acetylsalicylic prophylaxis	Nulliparity
	Family history of preeclampsia (mother and/or sisters)
	Age ≥ 35 years
	Previous pregnancy with adverse outcome (placental abruption, low birth weight at > 37 weeks, preterm labor)
	Interval > 10 years since last pregnancy

Considering the current evidence available and the epidemiological scenario of Rio de Janeiro state, the State Department for Health, based on the evaluation of its technical staff, decides to establish a universal public policy for the prediction and secondary prevention of preeclampsia by the following recommendations:

1. All pregnant women should be advised to practice physical activity to reduce the likelihood of developing gestational hypertension or preeclampsia, as long as there is no contraindication. Pregnant women should perform at least 140 minutes per week of moderate-intensity exercise, such as brisk walking, water aerobics, and stationary cycling, with moderate effort and resistance training. Once the diagnosis of preeclampsia is confirmed, physical activity needs to be adjusted and reduced to avoid harm to uteroplacental circulation.
2. All pregnant women from the state of Rio de Janeiro must be considered to belong to a population with low calcium intake. Therefore, it is recommended that everyone receive a supplement of 1,500mg of elemental calcium per day. Pregnant women should preferably take the tablets with some food, but not those rich in phytates, oxalates, or iron (for example, beans, liver, spinach, chard, kale, beetroot, or sweet potatoes), as these substances hinder the absorption of calcium. Furthermore, calcium supplements should be taken at least 2 hours apart from iron supplements or multivitamins containing iron, as this mineral reduces calcium absorption. It is recommended that calcium supplementation be started from 12 weeks of gestation (preferably before the 16th week and can be started up to the 20th week) and continued until the 36th week of gestation. Calcium supplementation should also be accompanied by encouragement to eat foods rich in calcium during pregnancy, especially milk and its derivatives, dark green vegetables, such as kale and broccoli, and some seafood, such as sardines.
3. Acetylsalicylic acid should be indicated for pregnant at risk of preeclampsia (1 high-risk marker or ≥ two moderate-risk markers). This medication should be started from the 12th week of gestation onwards (preferably before the 16th and can be started up to the 20th week of gestation) and continued until the 36th week of gestation. It should be administered at a dose of 100 mg and should be taken at night.
4. Suspension of calcium and acetylsalicylic acid is recommended if the diagnosis of preeclampsia is confirmed.

Although physical activity is a simple strategy, it is still a guideline that must be disseminated more widely during prenatal care. And, despite the evidence regarding the use of acetylsalicylic acid for risk groups being well-established for a decade, numerous lost opportunities in implementing this prevention modality are observed in clinical practice.

The great innovation of this public policy is the universal calcium supplementation, implemented for the first time on a population scale by a Brazilian state: the result of the integration of technical staff and political agents. Without raising awareness among health authorities about the severe problem of MM, there is no vision of a sustained change in this scenario. Therefore, academia must get closer to public management, offering clinical evidence and quality guidelines to guide the decision-making of those who have the power to change the direction of public health in our country.
